# *In Vitro* and *In Vivo* Effects of Metformin on Osteopontin Expression in Mice Adipose-Derived Multipotent Stromal Cells and Adipose Tissue

**DOI:** 10.1155/2015/814896

**Published:** 2015-04-30

**Authors:** Agnieszka Śmieszek, Katarzyna Basińska, Klaudia Chrząstek, Krzysztof Marycz

**Affiliations:** ^1^The Faculty of Biology and Animal Science, University of Environmental and Life Sciences, Kozuchowska 5b Street, 50-631 Wroclaw, Poland; ^2^Wrocławskie Centrum Badań EIT+, Stablowicka 147 Street, 54-066 Wroclaw, Poland

## Abstract

Metformin is applied not only as antidiabetic drug, but also in the treatment of obesity or as antiaging drug. The first part of the research discussed the effect of metformin at concentrations of 1 mM, 5 mM, and 10 mM on the morphology, ultrastructure, and proliferation potential of mice adipose-derived multipotent mesenchymal stromal cells (ASCs) *in vitro*. Additionally, we determined the influence of metformin on mice adipose tissue metabolism. This study has shown for the first time that metformin inhibits the proliferative potential of ASCs *in vitro* in a dose- and time-dependent manner. In addition, we have found a significant correlation between the activity of ASCs and osteopontin at the mRNA and protein level. Furthermore, we have demonstrated that 5 mM and 10 mM metformin have cytotoxic effect on ASCs, causing severe morphological, ultrastructural, and apoptotic changes. The reduced level of OPN in the adipose tissue of metformin-treated animals strongly correlated with the lower expression of Ki67 and CD105 and increased caspase-3. The metformin influenced also circulating levels of OPN, which is what was found with systemic and local action of metformin. The results are a valuable source of information regarding the *in vitro* effect of metformin on adipose-derived stem cells.

## 1. Introduction

Metformin (N,N′-dimethylbiguanide) is a widely used drug for the treatment of type 2 diabetes mellitus, condition associated with insulin sensitivity and obesity. Additionally, it has been shown that metformin may inhibit growth of tumor cells and thus potentially may find application in therapy of various types of cancer [1–6]. Metformin was also classified for conceptual group of drugs, known as calorie restriction mimetics (CRM). Anisimov et al. [7] have shown that calorie restriction model involving metformin supplementation is a very effective way of increasing the lifespan by reducing morbidity and mortality both of healthy mice and of mice with tumor.

The progressive aging of the human population together with increasing obesity are the main reasons of metabolic diseases [8]. The World Health Organization (WHO) reported that over 500 million people suffer from overweight and/or obesity (data from 2013). This serious medical condition becomes a great challenge for modern pharmacotherapy. Metformin is considered a part of the solution to this problem due to its multidirectional action on adipose tissue metabolism [9, 10]. Metformin has an ability to decrease adiposity and obesity related conditions what was proved either in human or animal model [11–14]. Additionally, according to a randomized study performed by Srinivasan et al. [13] and Yanovski et al. [14] children and adolescents may be much more responsive to metformin-induced weight reduction as compared to adults. These results might suggest that the metformin has an effect not only on morphology and distribution of fat tissue but also on proliferation potential of adipocytes precursors.

Progenitor cells in the uncultured stroma-vascular fraction (SVF) derived from adipose tissue usually amount to up to 3% of the whole cells. This unique population is defined as adipose-derived multipotent stromal cells, ASCs [15, 16]. Although ASCs are of mesoderm origin, these stem cells possess a unique ability to differentiate into ectoderm and endoderm lineages as well as mesoderm cells [17]. ASCs are increasingly being not only used as a research tool, but also applied in the human and veterinary medicine cell therapy. The reason for this is that they can be easily obtained in large quantities with little donor site morbidity or patient discomfort. ASCs become a source of cells characterized by high proliferative potential, stable growth, and kinetics and thus are considered a promising candidate in regenerative medicine. ASC-based therapies have been shown to be safe and efficacious in the preclinical and clinical studies of various injuries and diseases [18, 19].

Given the fact that metformin exerts an unquestionable influence on adipose tissue, which becomes a source of a unique stem cell population, it was reasonable to investigate the effects of this compound on the proliferation potential, morphology, and ultrastructure of the ASCs.

Markers that can be associated with cells proliferation are Ki-67 and osteopontin (OPN). Additionally, expression of another marker, for example, CD105/endoglin, should be taken into consideration, because it defines MSCs provenance and is associated with preadipocytes proliferation and differentiation potential [20, 21]. Ki-67 is a ubiquitous human nuclear protein expressed in G_1_, S, and G_2_ phases of the cell cycle [22, 23] and therefore is used as indicator of growth fraction of particular cells population. Ki-67 was also used to quantify preadipocyte replication when assessing cellular turnover within adipose tissue of mice and humans [24]. In turn, osteopontin is a multifunctional protein mainly associated with osseous tissue metabolism and bone remodeling [25]. It is expressed in proliferating fibroblast and in osteogenic and periodontal ligament cell populations [26, 27]. Moreover, OPN upregulation is also connected with variety of acute and chronic inflammatory conditions, such as wound healing, fibrosis, autoimmune disease, and atherosclerosis [28]. The osteopontin is also regarded as a mediator linking obesity to the development of insulin resistance by promoting inflammation and the accumulation of macrophages in adipose tissue [29].

Bearing in mind all mentioned facts, determination of expressions of Ki-67 and osteopontin in adipose tissue of mice treated with metformin seems to be crucial regarding its influence on ASCs proliferation activity. To the best of our knowledge, this issue has never been investigated previously. Therefore, to obtain complex information concerning effect of metformin of ASCs and adipose tissue, we decided to approach this subject bidirectionally, performing* in vitro* and* in vivo* studies. Analysis of cytotoxic effect of metformin was performed on ASCs isolated from mice. We evaluated not only proliferative activity of ASCs exposed to metformin, but also their morphology and ultrastructure. Additionally we investigated the OPN gene expression and protein secretion.

Metformin accumulates in tissues of diabetic mice in concentrations several times higher than those in blood [30, 31], and therefore three concentrations of metformin (1 mM, 5 mM, and 10 mM) were applied in this study.

The aim of* in vivo* study was to evaluate the effect of metformin on histological structure of adipose tissue and to determine expression of proliferation markers. We believed that the data presented in this study will provide meaningful information of clinical significance, especially due to the fact that substantial part of our research was dedicated to cells morphology investigation, important in diagnosis of pathology.

## 2. Material and Methods

All reagents used in this experiment were purchased from Sigma-Aldrich (Poland), unless indicated otherwise.

All experimental procedures were approved by the II Local Ethics Committee of Environmental and Life Science University* (Dec. No. 177/2010 of 11.15.2010)*.

### 2.1. *In Vitro* Study

#### 2.1.1. Isolation of Mesenchymal Stromal Cells from Adipose Tissue

The cells were isolated from subcutaneous adipose tissue of six C57BL/6 mice using protocol established previously [32, 33]. Material was collected from the abdominal region of each animal. Tissue fragments were minced and digested using type I collagenase for 40 minutes at 37°C. Next, the tissue homogenates were centrifuged for 10 min at 1200 ×g. The supernatants were aspirated and the pellets of the stromal-vascular adipose fraction, containing MSCs precursors, were washed with Hank's balanced salt solution and centrifuged three times (5 min at 600 ×g). Pellets were then suspended in Dulbecco's modified Eagle's medium (DMEM) with Nutrient F-12 Ham and transferred to culture flasks. Cultures were maintained at 37°C in a humidified atmosphere of 5% CO_2_ and 95% air. The cells were passaged after reaching 80–90% confluence. Subsequent cultures were propagated in DMEM containing 4500 mg/L of glucose. Culture media were supplemented with 10% FBS and 1% antibiotics (penicillin and streptomycin). The medium was changed every two days. Before experiment, the cells were passaged three times, using trypsin solution (TrypLE; Life Technologies) according to manufacturers' instruction.

#### 2.1.2. Phenotypic Characterization of Isolated Cells and Multipotency Assay

Phenotypic characterization of cells was performed using ASCs cultures derived from animals assigned for* in vitro* tests. Analyzed cells were adjusted in terms of subculture (all after second passage). For staining, cultures were maintained in 24-well dishes designated for immunofluorescence preparations (Sarstedt). For each staining, cultures were run in triplicate. To determine the phenotype of cells, the expression of following markers was investigated: CD29, CD44, CD45, CD73, and CD105. The antibodies used for the analysis were purchased from Sigma (rabbit anti-mouse integrin-b-1 (CD29), dilution 1 : 100; rabbit anti-mouse NT5E (CD73), dilution 1 : 200; rabbit anti-mouse endoglin (CD105), dilution 1 : 200; rabbit anti-mouse CD45, dilution 1 : 100), except for anti-CD44 which was obtained from R&D Systems (rabbit anti-mouse CD44, dilution 1 : 100). Secondary antibody conjugated with atto-488 fluorescence label was purchased from Sigma (goat anti-mouse IgG atto-488, dilution 1 : 400). Immunocytochemistry was performed using the general protocol described by the manufacturer. Incubation with primary antibodies was carried out overnight, while the reaction with secondary antibodies was performed at 37°C for 1 hour in the dark. In order to determine background fluorescence and exclude nonspecific staining, negative controls were included to assess the binding specificity of secondary antibodies. Negative controls were incubated with secondary antibody for 1 hour at 37°C in the dark. Additionally, specimens were counterstained with 4′,6-diamidino-2-phenylindole (DAPI) to visualize the nuclei. The samples were imaged using an inverted fluorescence microscope (AxioObserverA1, Carl Zeiss, Jena, Germany). Images were captured with Cannon PowerShot camera and merged using AxioVision 4.8 software (Carl Zeiss, Jena, Germany).

#### 2.1.3. Determination of Multipotent Character of Mice ASCs

Osteogenic and adipogenic differentiation of ASCs were induced using commercial kits (StemPro, Life Technologies). Cultures under standard and osteo- and adipogenic conditions were maintained in 24-well dishes. Experiments were carried out simultaneously, each in triplicate according to the previous findings [33]. Osteogenesis was induced during 21-day period, while stimulation toward adipocytes lasted for 14 days. To evaluate results of osteogenic and adipogenic differentiation, two specific staining methods were used, that is, Alizarin Red for determination of calcium deposits and detection of neutral lipid deposits performed using HCS LipidTOX Green neutral lipid stain, and according to the manufacturers' instruction preparations were analyzed using Axio Observer A1 inverted and epifluorescent microscope (Axio Observer A1, Carl Zeiss, Jena, Germany), while the documentation was made using Cannon PowerShot camera.

#### 2.1.4. ASC Proliferation Assay

For assays, cells were seeded in 24-well plates at a concentration equal to 3 × 10^4^. Cells were suspended in 0.5 mL of culture medium per well. Metformin hydrochloride (Metformax 850, Teva Pharmaceuticals, Poland) was crushed in a mortar and dissolved in the culture medium at the following concentrations: 1 mM, 5 mM, and 10 mM. Nontreated cells served as a control for comparison with the test culture. In order to performe further analysis (including morphology, ultrastructure, and gene expression), each culture was performed in six replications. Proper number of culture plates was performed to investigate cells in the chosen time-points. The first dosage of metformin was added to the culture after 24 hours. During the cell treatment medium was changed every day. Culture media were collected for determination of protein level. The viability of the cells was evaluated after 24, 48, and 72 hours using a resazurin-resorufin system (Alamar Blue). To perform the test, culture media were removed and replaced with a medium containing 10% of the dye. Cells were incubated with the dye in the CO_2_ incubator for 2 hours. The supernatants were collected and transferred to a 96-well microplate reader (Spectrostar Nano, BMG Labtech). The absorbance of the supernatants was measured spectrophotometrically at a wavelength of 600 nm for resazurin and 690 nm as a reference wavelength. Each measurement included a blank sample, containing complete medium without cells. Proliferation activity was described using ΔΔ*A* value, expressing difference of absorbance of supernatants at 600 and 690 nm and including absorbance of blank samples.

#### 2.1.5. Examination of Morphology, Viability, and Ultrastructure of ASCs

ASC morphology was evaluated under epifluorescent microscope (Zeiss, Axio Observer A1) and scanning electron microscope (SEM, Zeiss Evo LS 15). The analysis of morphology was performed after 24 h and 72 h of culture in 24-well plates. The preparation of ASCs for fluorescent microscopy was performed according to established procedure [34, 35] and included the following stages: (i) washing three times using HBSS, 1 min each; (ii) fixation of cells in 4% ice cold paraformaldehyde, performed overnight at 4°C; (iii) washing of cells (as described above); (iv) 15 min of cell permeabilization with 0.1% Triton X-100 at room temperature; (v) washing of cells (as described above); (vi) staining with atto-488-labeled phalloidin (1 : 800) for 30 min performed in the dark at room temperature; (vii) counterstaining using diamidino-2-phenylindole (DAPI; 1 : 1000) for 5 min at room temperature. For the detection of apoptotic cells, simultaneously to DAPI staining, immunofluorescence staining of caspase-3 was performed using polyclonal rabbit anti-mouse caspase-3 antibody (dilution 1 : 100). Additionally, for detection of dead and apoptotic cells analysis using Cellstain Double Staining Kit with propidium iodide was conducted. Staining procedure was performed according to the protocols available from the manufacturers. Images were captured using Cannon PowerShot Camera.

For SEM analysis, (i) cells were fixed in 2.5% glutaraldehyde in DMEM, (ii) rinsed with HBSS, (iii) dehydrated in a graded ethanol series (from 50% to 100%, increasing every 10%), (iv) air-dried for 30 min at room temperature, and (v) coated with gold using 300-second program (Edwards, Scancoat six). Prepared samples were captured using a SE1 detector at 10 kV filament tension (SEM, Zeiss Evo LS 15) under 500x and 5000x magnification.

For TEM analysis, we used procedure described previously [36]. Briefly, cells were fixed overnight at 4°C in 2.5% glutaraldehyde in DMEM. After fixation, cells were centrifuged at 2000 ×g for 10 min and rinsed with PBS (0.1 M, pH = 7.0) for 30 min at room temperature. After washing, cells were centrifuged again using parameters provided above. Pellets were incubated with 1% osmium tetroxide in PBS for 2 hours. Cells were washed once again using 0.1 M PBS and centrifuged. Following this procedure, the cells were dehydrated in a graded acetone series (30–100%) and embedded using Agar Low Viscosity Resin Kit (Agar Scientific Ltd., Stansted). Ultrathin sections (80 nm) of the specimens were collected on copper grids. Cells were contrasted with uranyl acetate (30 min incubation) and lead citrate (15 min incubation). Cells were imaged with TEM detector, at 10 kV filament tension.

#### 2.1.6. Analysis of Gene Expression: Real-Time Reverse Transcription Polymerase Chain Reaction (qRT-PCR)

After 24 and 72 hours of culture, cells were rinsed twice using HBSS and then homogenized using 0.8 mL of TRI Reagent. Procedure of cells' homogenization was performed directly in the culture dish. Preparations were obtained from samples prepared in duplicate. Total RNA was isolated using a single-step method described by Chomczynski and Sacchi [37]. The resulting samples were diluted in DEPC-treated water. The quantity and quality of total RNA were determined using a nanospectrophotometer (VPA biowave II). Traces of genomic DNA (gDNA) were digested with DNase I RNase-free kit (Thermo Scientific). Each reaction contained 100 ng of total RNA. Complementary DNA (cDNA) was obtained in the reaction with Moloney Murine Leukemia Virus Reverse Transcriptase (M-MLV RT) and oligo(dT)15 primers (Verte KIT oligo(dT)15, Novazym). Both RNA purification and cDNA synthesis were performed in accordance with manufacturers' instructions using the T100 Thermal Cycler (Bio-Rad). Detection of osteopontin was performed using following primers: (i) forward 5′-AGACCATGCAGAGAGCGAG-3′ and (ii) reverse 5′-GCCCTTTCCGTTGTTGTCCT-3′ (NCBI accession number: NM_001204203.1). Beta-2 microglobulin (*β*2M) was used as housekeeping gene; sequences of primers were as follows: (i) forward 5′-CATACGCCTGCAGAGTTAAGCA-3′ and (ii) reverse 5′-GATCACATGTCTCGATCCCAGTAG-3′ (NCBI accession number: NM_009735.3). Quantitative RT-PCR was carried out in a total volume of 20 *μ*L using SensiFast SYBR and Fluorescein Kit (Bioline). The concentration of primers in each reaction was 500 nM. The following cycling conditions were applied: 95°C for 2 min, followed by 45 cycles at 95°C for 5 sec, annealing 58°C for 10 sec, and 72°C step for 5 sec with a single fluorescence measurement. Analysis of the dissociation curve of amplicons was performed to determine the specificity of the PCR products. Melting curve was determined with a gradient program of the range from 65 to 95°C at a heating rate of 0.2°C/s and continuous measurement of the fluorescence. Reactions were performed in three repetitions. The values of the threshold cycle (Ct) obtained in each experiment were used to calculate fold change in relation to the expression of housekeeping gene. Real-time PCR was performed using CFX Connect Real-Time PCR Detection System (BioRad).

#### 2.1.7. Osteopontin Detection in Culture Supernatants

The osteopontin level was determined in supernatants collected after experimental cultures. For analysis, each culture medium was prepared in duplicate. The concentration of OPN was measured by enzyme-linked immunosorbent assay (ELISA) using a commercially available ELISA kit (DuoSet ELISA Development kit; R&D Systems). ELISA was performed according to the manufacturer's instructions. For the analysis, supernatants were diluted 5 times. All tested samples and standards were measured in two replicates.

### 2.2. *In Vivo* Study

#### 2.2.1. Animals and Subcutaneous Tissue Collection

Eighteen C57BL/6 mice (females, 4-week-old) were housed three per cage in an ultraclean facility on ventilated racks and were provided food and water* ad libitum* during the 5-week experiment. The animals were purchased from Animal Laboratory House, Wroclaw Medical School, and housed in the Animal Experimental Laboratory (Wroclaw Medical School, Poland). Mice received a standard diet with 4,2% fat (Morawski, Labofeed H, Poland) and were maintained on a 12-hour light-dark cycle at 22 ± 0.2°C.

The animals used in the study were divided into two groups: (i) control (*n* = 9) receiving drinking water only and (ii) the group receiving metformin in drinking water at a concentration equal to 2,8 mg/day (Metformax 850; Teva Pharmaceuticals, Poland) (*n* = 9). Water was changed every two days.

After the experiment, mice were fasted for 24 h. Body weight measurement was carried out using electronic weight (RADWAG PS/C1 series, Poland). Blood was collected from animals using cardiac puncture method. Biochemical analysis of blood samples, such as glucose and lipids measurements, was performed with Erba XL 300 platform (Erba Diagnostics Mannheim GmbH, Germany). After euthanasia of animals, adipose tissue was collected from abdominal subcutaneous layer of all animals.

#### 2.2.2. Histology and Immunohistochemistry

Subcutaneous adipose tissue (0.5 g) was fixed in a cold 4% paraformaldehyde (Sigma-Aldrich). Next, samples were transferred to 0.1 M phosphate buffer and incubated for 24 hours. The samples were then treated with 18% sucrose for three days. The samples were applied on a specimen holder using tissue freezing medium and transferred to a freezer (−20°C). The samples were cut into sections of 0.5 *μ*m thickness and subsequently stained using Mayer hematoxylin (Sigma-Aldrich). Sections were prepared in six replicates and deposited on Superfrost Ultra Plus Adhesion Slides (Thermo Scientific).

Morphometric analysis of adipose tissue was performed with Axio Imager light microscope (Zeiss) using 10 fields of view for each individual (each slide) based on our previous experience [38]. Histological photographs were compiled using an Axio Camera (Zeiss).

The Image J software (http://rsbweb.nih.gov/ij/) was used to measure size and area of adipocytes. Adipocytes that were damaged or overly distorted owing to processing were not included in the measurements. Moreover, number of crown-like structures (CLS) was determined from six random microscopic fields magnified 100x.

For immunohistochemistry, 4 *μ*m frozen sections were placed on Superfrost Ultra Plus Adhesion Slides (Thermo Scientific). The samples were stored at −20°C and before preparations were rehydrated. For each staining, two specimens were prepared. Antibodies used in the experiment were raised against mouse OPN, Ki67 (1 : 200) (both purchased from Abcam), and caspase-3 markers (1 : 100) (Sigma) as well as CD44 (1 : 500) and CD105 (1 : 100) antigens (both from Abcam). Obtained samples were incubated with specific antibodies over night at 4°C. After rinsing with HBSS, sections were incubated for 1 hour with goat anti-rabbit IgG antibody conjugated with atto-488 (dilution 1 : 200), avoiding direct light. Nuclei were counterstained using 4′,6-Diamidino-2-Phenylindole (DAPI, dilution 1 : 1000). Incubation of specimens with DAPI (5 min) was followed by HBSS washing. Slides were mounted using Fluoromount to image the sections. Specimens were visualized using epifluorescence microscopy (Axio Observer A1, Zeiss) with a 100-fold magnification. Images were saved and processed using Axio Imager 4.7 software. Nonspecific staining of the samples was reduced by incubation with a blocking buffer for 30 minutes. The incubation buffer contained goat serum (1 : 100), bovine albumin (1 : 10), and Triton-X (0.3%).

#### 2.2.3. Detection of OPN in Mice Serum

The OPN concentration was measured using ELISA kit (DuoSet ELISA Development kit; R&D Systems). Serum was obtained from all control and experimental animals. The test was performed similarly as it was described in paragraph 1.7, with the difference that serum samples required 100-fold dilution. All samples and standards were measured in duplicate.

### 2.3. Statistical Analysis

The normality of the population data was determined using Shapiro-Wilk test, while equality of variances was assessed by Levene's test. Differences between groups were determined using one- or two-way analysis of variance (ANOVA). Statistical analysis was performed with STATISTICA 10.0 software (StatSoft, Inc., Statistica for Windows, Tulsa, OK, USA). Differences with a probability of *P* < 0.05 were considered significant.

## 3. Results

### 3.1. *In Vitro* Study

#### 3.1.1. Phenotypic Characterization of Isolated ASCs and Multipotency Assay

**Figure 1:**
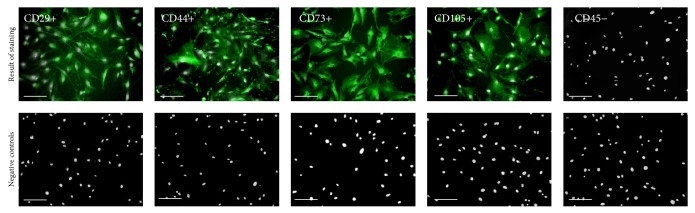
The results of mice adipose-derived mesenchymal stem cells immunophenotyping represented in images. Immunostaining analysis was performed on three ASCs cultures, after second passage. The obtained populations of cells were positive for markers characteristic for mesenchymal cells, that is, CD29, CD44, CD73, and CD105, and were negative for hematopoietic marker CD45. Particular markers were stained with specific primary antibody and secondary antibody conjugated with atto-488 (positive reactions shown in green), and nuclei stained with DAPI are shown in white. Results of negative staining are included in the Figure. Magnification used is 100x. Scale bar = 250 *μ*m.

**Figure 2:**
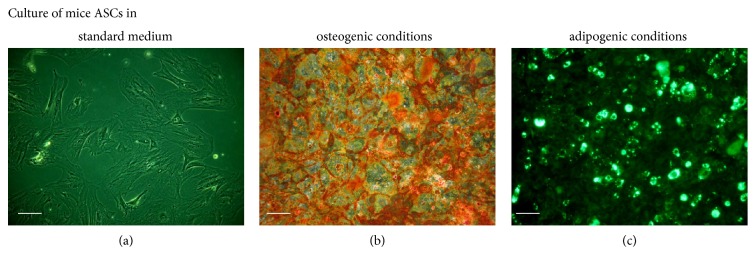
The morphology of adipose-derived mesenchymal stem cells isolated from subcutaneous fat tissue of mice in standard (a), osteogenic (b) and adipogenic culture (c). The calcium deposits formed in extracellular matrix of osteogenic culture were stained using Alizarin Red. The lipid-rich cellular organelles formed after adipogenic stimulation are visible as green droplets. Images included in the graph were chosen as representative. Both control and experimental cultures were carried out in triplicate. Magnification used is 100x. Scale bar is 200 *μ*m.

To confirm multipotent and mesenchymal character of isolated stromal cells, immunohistochemical staining and multipotency assay were performed. Analyses were conducted with regard to the recommendations of International Society of Cellular Therapy [39, 40]. Immunohistochemical analysis revealed that ASCs showed a negative reaction with an antibody against the hematopoietic marker, CD45 (Figure 1). In contrast, the cells were positive for mesenchymal markers characteristic for multipotent stromal cells, for example, CD29, CD44, CD73, and CD105 (Figure 1). The multipotency of mice adipose-derived mesenchymal stem cells was confirmed by the positive results of osteo- and adipogenic differentiation (Figure 2). ASCs cultured under osteogenic conditions formed extracellular matrix rich in mineral calcium deposits visualized by Alizarin Red staining (Figure 2(b)). In adipogenic cultures after 14 days of stimulation the formation of lipid droplets was observed (Figure 2(c)).

#### 3.1.2. Proliferation Activity (PA) of Adipose-Derived Stem Cells* In Vitro*

**Figure 3:**
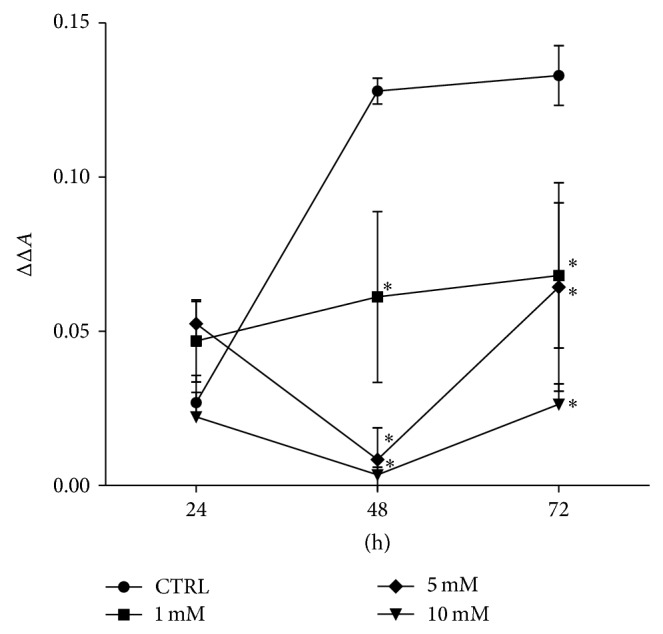
Proliferation activity of ASCs after metformin treatment at concentrations of 1 mM, 5 mM, and 10 mM in comparison to the control culture (CTRL). The *X*-axis refers to the time of cells' propagation. The difference between absorbance read at 600 nm and 690 nm, including absorbance of blank sample, was indicated as ΔΔ*A* mark (*Y*-axis). The statistical significance (*P* < 0.01) was indicated with asterisk (∗). Error bars represent standard deviation from the mean value calculated from three separated measurements.

The proliferation activity of ASCs* in vitro*, after exposition to the investigated concentrations of metformin, was evaluated after 24, 48, and 72 hours of culture (Figure 3). Determination of cell activity in control culture demonstrated that during the first 48 h the growth of cells was exponential. Between 48 and 72 hours of the culture, the growth rate declined, implying stationary phase of growth. Proliferation of ASCs cultures significantly decreased after 48 h of exposition to the metformin. The inhibition of ASCs cell growth by metformin occurred in a dose-dependent manner. Despite the fact that proliferation rate of ASCs in cultures with metformin at concentration equal to 1 mM was significantly lower in relation to the control culture, the growth curve had exponential character. Exposure of ASCs to the 5 mM and 10 mM metformin inhibited growth of cells; however after 72 h the proliferation activity of cultures was restored but at the level significantly lower than in control cultures.

#### 3.1.3. The Morphology and Ultrastructure of ASCs Treated with Various Concentrations of Metformin

**Figure 4:**
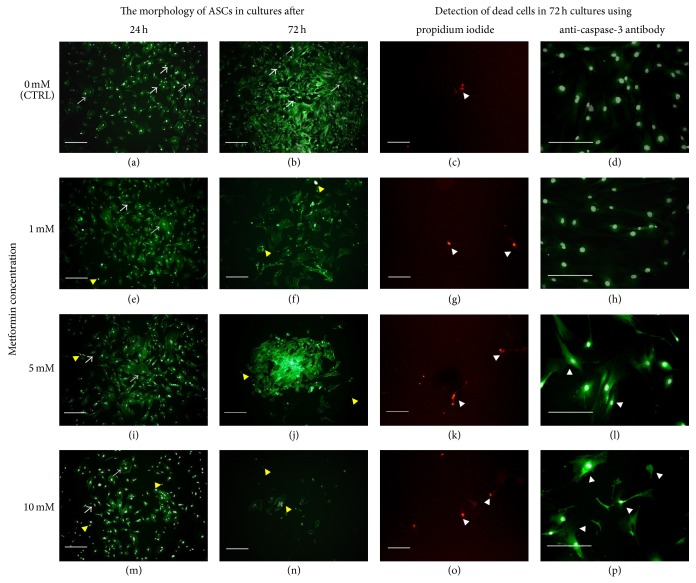
The morphological changes of ASCs after 24 and 72 hours in the cultures treated with 1 mM ((e) and (f)), 5 mM ((i) and (j)), and 10 mM ((m) and (n)) of metformin in comparison to the control cultures ((a) and (b)). The graphs provided are representative, showing characteristic features of investigated cultures. The ASCs cultures were characterized by the presence of small (representative indicated with thin arrows) and larger cells (representative indicated with thick arrows). Actin cytoskeleton was visualized using atto-488 phalloidin (green-stained cell bodies), while nuclei were visualized with DAPI stating (white dots). Apoptotic bodies occurred in cultures were indicated with yellow arrowheads, while dead cells were indicated with white arrowheads. Dead cells identified with propidium iodide are red-stained, while caspase-3 positive cells are green-stained. Magnification used is 50x, and scale bar is 200 *μ*m.

The morphological and ultrastructural changes of ASCs were examined after 24 and 72 h of the experiment in cultures treated with 1 mM, 5 mM, and 10 mM metformin and compared to the control culture (Figures 4, 6, and 7). The characteristic feature of the obtained ASCs cultures was occurrence of both small and large multipolar fibroblast-like cells (indicated in Figure 4 with white arrows). Analysis performed using epifluorescence microscope showed that exposure to metformin influenced cells morphology and growth pattern of cultures. In cultures treated with metformin, the large cells were more noticeable. The formation of apoptotic bodies was also more evident and their number was positively correlated with the metformin dose; the increase of apoptotic bodies, visualized with DAPI, was the most significant in culture exposed to 10 mM metformin. The ASCs maintained in control cultures were characterized by the high proliferative activity of cells also expressed by the formation of well-developed monolayer. Cultures treated with metformin were characterized by the irregular pattern of growth, both dispersed, as in cultures exposed to 1 mM metformin, and aggregated, as in culture treated with 5 mM of metformin. The degeneration of ASCs culture was observed after 72 h as a result of exposure to 10 mM of metformin.

**Figure 5:**
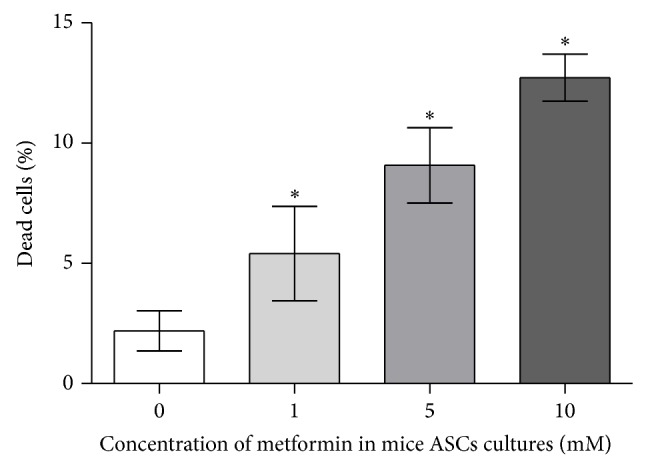
The percentage of dead cells, detecetd using Cellstain Double Staining Kit after 72 h of ASCs culture treated with 1 mM, 5 mM, and 10 mM of metformin in comparison to the control culture (0 mM of metformin).

Moreover, the analysis of characteristic signs of cell death, performed after 72 h of culture, showed that the percentage of dead and apoptotic cells was increasing with the metformin dose (Figure 4: propidium iodide and caspase-3 staining). Additionally, dead cells visualized with propidium iodide were counted. The result of comparative analysis regarding the number of dead cells in experimental versus control culture was shown in Figure 5.

**Figure 6:**
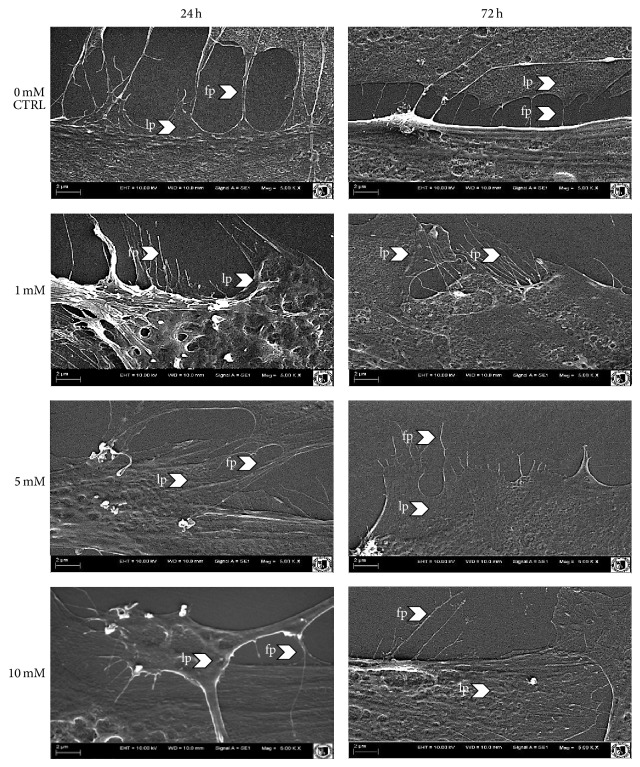
Analysis of cell surface morphology derived from experimental and control culture after 24 h and 72 h of propagation. Cellular projections were indicated with arrows (LP: lamellipodia and FP: filopodia). Depicted images are representative and were obtained from cultures carried out in duplicate.

Analysis of cellular junctions formation showed that addition of metformin caused decline of cytoplasmic projection. In the control culture, intercellular connections during the culture period became narrowed, indicating on tight cell-cell interaction in formed monolayer. Interactions between plasma membranes of cells in experimental cultures were noticeable only after 24 hours; however connections between cells were not maintained in cultures treated with the drug for 72 hours. Week development of cellular projections was evident especially when 5 mM and 10 mM metformin were used (Figure 6).

**Figure 7:**
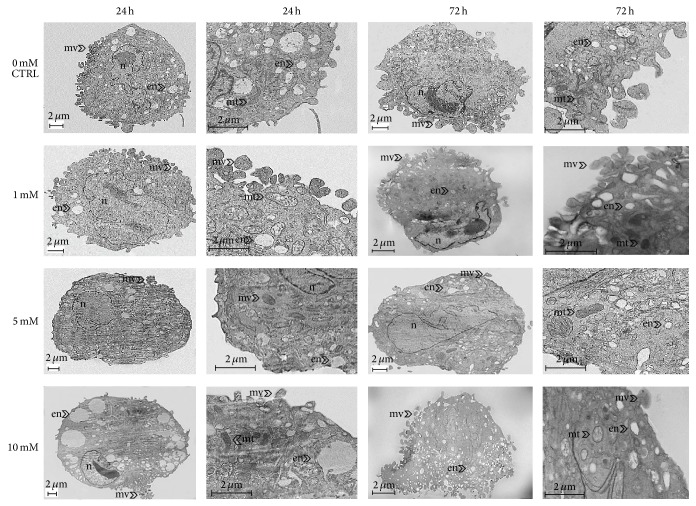
The ultrastructural changes of mice ASCs after 24 and 72 hours in the cultures treated with 1 mM, 5 mM, and 10 mM of metformin in comparison to the control culture. Typical ultrastructural features were indicated with proper abbreviations: nu: nucleus, en: endosomes, mv: microvesicles, and mt: mitochondria. Depicted images are representative, and were obtained from cultures carried out in duplicate.

The influence of metformin on cells' ultrastructure was expressed mainly by the changes of nuclei shape, size of endosomes and mitochondria, and the number of plasma membrane-derived particles (microvesicles, MVs) shedding (Figure 7). The ultrastructure of ASCs treated with the lowest concentration of metformin was comparable to the ultrastructure of control cells. Cells both derived from control culture and treated with 1 mM of metformin were characterized by the centrally located nuclei surrounded mainly by small, early endosomes and formation of MVs. Cells treated with 5 mM and 10 mM of metformin were characterized by peripherally located nuclei and enlarged, late endosomes. In these cultures, MVs shedding was incidental. Additionally, the ASCs treatment with metformin at concentrations of 5 and 10 mM caused ultrastructural abnormalities in mitochondria (enlargement of mitochondria). Similarly, after 72 h of culture control cells from control culture and from culture stimulated with 1 mM of metformin shared some common ultrastructural features, like peripheral displacement of nuclei and increased number of MVs. The distinctive feature of cells derived from culture treated with 5 mM was increase of nuclei size, occupying substantial part of cell body, and alteration of its shape. Additionally, the enlargement and bulging of mitochondria were maintained. Ultrastructural organization of ASCs treated with 10 mM was disrupted. Degenerative changes of ASCs, treated with the highest dose of the drug, involved invagination and disintegration of cellular membrane, excessive accumulation of endocytic vesicles, and disintegration of cell nuclei (Figure 7).

#### 3.1.4. Detection of OPN at the mRNA and Protein Level in ASC Cultures

**Figure 8:**
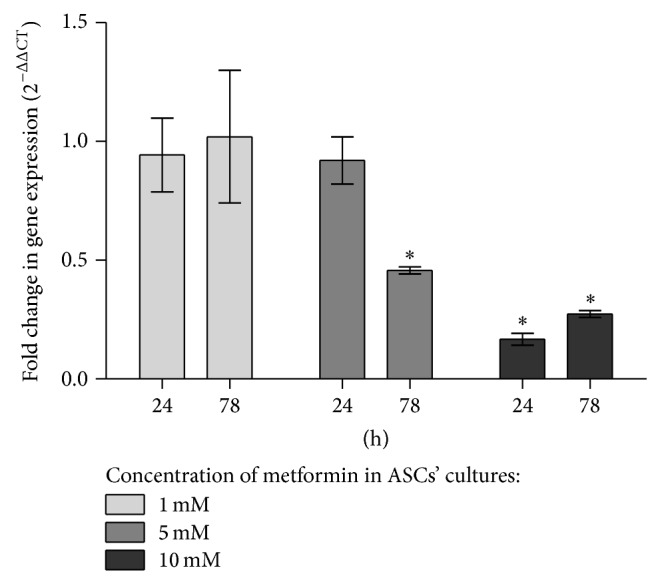
Expression of mRNA for OPN in experimental cultures. Quantification of relative values was performed using 2^−ΔΔCT^ method, normalizing data to control culture and including expression of reference gene. Data are presented as the mean fold change of relative expression and compared to control cultures (normalized to 100%); error bars represent standard deviation from the mean calculated for normalized values obtained in three separate reactions; asterisks represent statistically significant differences (*P* < 0.05).

Analysis of OPN gene expression was investigated in cells derived from experimental and control cultures, after 24 h and 72 h of propagation (Figure 8). Quantitative analysis of transcripts revealed that the expression of OPN in ASCs after 24 h treated with 1 mM and 5 mM of metformin decreased in comparison to the OPN mRNA expression in control culture; however observed differences were not statistically significant. The propagation of ASCs with 10 mM metformin for 24 h caused significant inhibition of OPN mRNA expression. Expression of OPN mRNA level was elevated after 72 h, when treated with 1 mM; however the increase was not statistically significant. Inhibition of OPN mRNA expression at significant level was noted in cultures maintained for 72 h with 5 mM and 10 mM of metformin.

**Figure 9:**
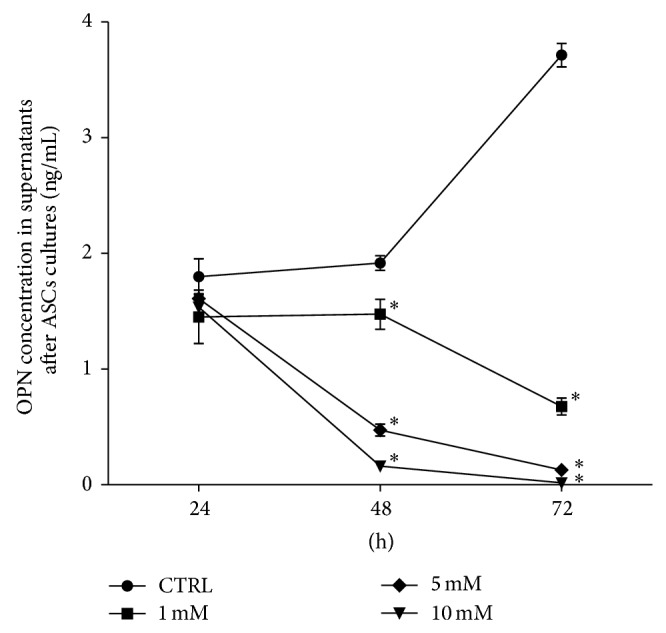
The mean concentration of OPN protein in the control (CTRL) and experimental cultures (1 mM, 5 mM, and 10 mM metformin) determined in supernatants collected at specific time of cultures (*X*-axis). The statistically significant decrease of OPN protein was shown at 48 h and 72 h after treatment with 5 mM and 10 mM and in all experimental groups, respectively. The statistically significant difference determined in relation to the results obtained for control group was indicated with asterisks (^∗^ *P* < 0.05). The mean values of OPN concentration were calculated from the results obtained in two measurements.

The level of secreted metformin in experimental cultures decreased in time and in dose-dependent manner, while in control cultures highest concentration of OPN protein was noted after 72 h of culture. The concentration of OPN decreased significantly in all experimental cultures after 48 h of propagation (Figure 9).

### 3.2. *In Vivo* Study

#### 3.2.1. Clinical Characterization of Animals

In our experiment, administration of metformin caused significant weight loss in C57BL/6 mice. Additionally, mice treated with metformin had lowered glucose and triglyceride levels, whereas cholesterol level increased (Table 1). All differences observed were statistically significant (*P* < 0.001).

#### 3.2.2. Morphology and Morphometry of Adipocytes

**Figure 10:**
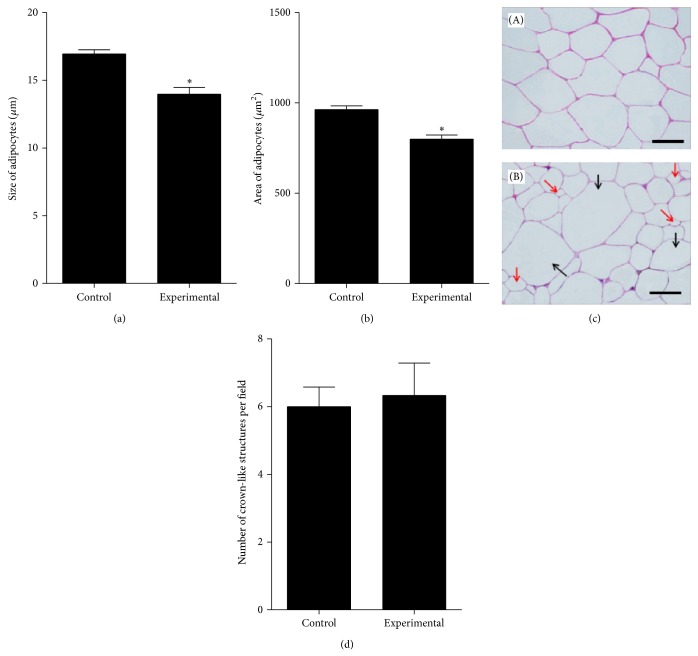
The results of morphometric analyses: comparison of the adipocytes size (a) and areas (b) in tissue of control and experimental group. Differences in cells dimensions were statistically significant at *P* < 0.05 (∗). Morphometric analysis of adipocytes was performed on six sections separately. Calculations were expressed as a mean value (*Y*-axis). (c) The results of hematoxylin-eosin (H-E) staining of adipose tissue sections derived from control group (A) and experimental group (B). Diversity of adipocytes in tissue of mice treated with metformin was indicated with arrows: red is indicating cells smaller in size and black is indicating cells of larger size. Scale bar is 75 *μ*m. (d) The results of crown-like structures determination performed on six different microscopic images. Crown-like structures are visible in sections as aggregated macrophages accumulated and fused around adipocytes.

Evaluation of the adipocytes morphology and results of morphometric analyses are shown in Figure 10. Histological examination of abdominal subcutaneous fat tissue collected from mice treated with metformin and from control animals revealed significant morphological changes regarding size of cells and their organization in the tissue. Adipocytes forming adipose tissue of control mice were larger (16.95 ± 1.21 *μ*m) than adipocytes of experimental mice (13.97 ± 2.01 *μ*m). The areas of adipocytes derived from experimental animals were also smaller than in control group. Additionally, population of adipocytes in tissue of control mice was more homogenous both in size (within a range of 15.46–18.67 *μ*m) and in shape (mainly polygonal), while adipocytes forming adipose tissue of experimental mouse had divergent size (within a range of 11.02–18.21 *μ*m) and morphology. The formation of crown-like structures in adipose tissue of experimental animals was more evident; however comparative analysis of CLS number showed that difference between groups is not relevant.

#### 3.2.3. Fluorescence Immunohistochemistry

**Figure 11:**
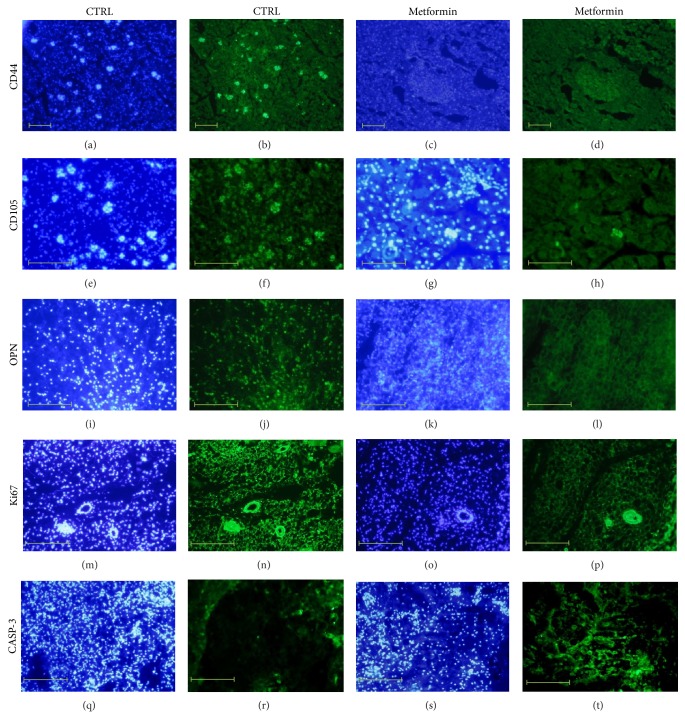
Results of immunohistochemical staining for CD44, CD105, OPN, Ki67, and caspase-3 (CASP-3). In order to recognize the nuclei location, the images of specific staining were arranged with images presenting results of DAPI staining (blue-tone images). The weaker CD44^+^, CD105^+^, OPN^+^, and Ki67^+^ reactions were noted in subcutaneous fat tissues collected from experimental group. Stronger positive reaction for caspase-3 was seen in fat biopsies derived from animals treated with metformin than in control group. Immunostaining reactions were prepared in duplicate. Magnifications are 50x and 100x; scale bar is 400 *μ*m.

The immunohistochemical analysis of subcutaneous fat tissue collected from animals treated with metformin and control animals demonstrated positive reaction for the following markers: CD105, CD44, OPN, Ki67, and caspase-3 (CASP-3). However, substantial differences in the quality and intensity of reaction between investigated groups were observed; results were presented in Figure 11. Fat biopsies of control animals exhibited stronger reaction for CD44^+^ and CD105^+^(Figures 11(b) and 11(f), resp.) in comparison to animals treated with metformin (Figures 11(d) and 11(h)). The expression of OPN in the fat tissue of animals treated with metformin was weaker (Figure 11(l)) when compared to the control animals (Figure 11(j)). The reaction with Ki67 antigen was also more abundant in the fat biopsies of control animals (Figure 11(n)) than in tissues obtained from experimental animals (Figure 11(p)). In turn, the expression of caspase-3 staining in the fat tissue of animals treated with metformin was higher (Figure 11(t)), when compared to the tissue derived from control animals (Figure 11(r)).

#### 3.2.4. Determination of OPN Level in Serum

**Figure 12:**
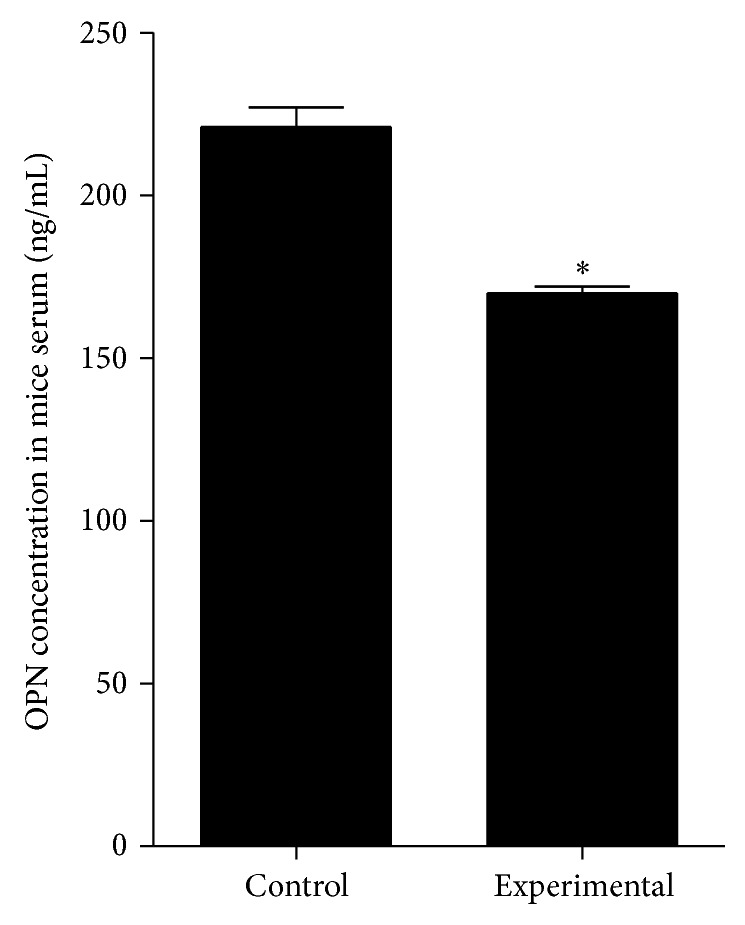
The concentration of OPN protein in the serum of mice derived from control and experimental-metformin treated group. Mean values express osteopontin level of nine mice belonging to the investigated group. The statistically significant decrease of OPN protein was noted in serum of experimental animals. The statistically significant difference determined in relation to the results obtained for control group was indicated with asterisks (^∗^ *P* < 0.05).

Evaluation of OPN serum concentration measured with ELISA showed that mice treated with metformin exhibit significantly lower OPN level than mouse in control group. The results of statistical analysis are presented in Figure 12.

## 4. Discussion

In the present study we investigated the effects of metformin on adipose-derived mesenchymal multipotent stromal cells (ASCs) and adipocytes, especially in relation to their proliferative potential. We have also focused on the evaluation of the influence of metformin on the morphology of both cells population of adipose tissue, and thus the results presented here include* in vitro* and* in vivo* studies. Metformin is known mainly for its antidiabetic properties, but it may also produce physiological effects comparable to calorie restriction. For instant, it may decrease fat mass and reduce size of adipocytes, as it was shown in depots of subcutaneous tissue of patients with diabetes [41, 42]. Additionally, it has been shown in* in vitro* studies that metformin may increase glucose uptake of adipocytes and embryonic tissues [43, 44]. Because glucose is a crucial nutrient for proliferating cells, it was of our interest whether metformin has an effect on proliferation of adipocytes precursors (ASCs) and adipocytes. The application of ASCs for regenerative medicine becomes more and more frequently investigated in both basic biological research, fundamental in context of establishing of ASCs cytophysiology, and preclinical studies [45].

As it was previously shown, metformin may inhibit proliferation of stromal cells—fibroblasts [46] and cancer cells by activation of AMPK and inhibition of multiple molecular signaling pathways, which are important in protein synthesis control [47–49]. The field of our interest was to evaluate the influence of metformin on the expression of osteopontin which is a multifunctional protein involved in various inflammatory processes, cell proliferation and migration, and tissue remodeling [50].

When comparing proliferation activity of ASCs* in vitro* in experimental and control cultures with the supernatant concentration of OPN measured with ELISA, we have found similarity in the pattern of curves' course. In the first 24 hours of cultures the proliferation activity of cells and OPN concentration in all investigated groups were at the same level. Both proliferation and OPN concentration significantly decreased after 48 h in cultures exposed to the metformin. Inhibition of proliferation activity of cells was also correlated with OPN decrease at mRNA level; the transcript level depends on dose used in culture, and the lowest expression of OPN mRNA was noted in cultures treated with 10 mM of metformin.

Inhibited expression of OPN gene along with other osteogenic markers after treatment with metformin was noted previously in primary osteoblasts and mouse osteoblastic cell line (MC3T3-E1) [51]. In this paper, the authors revealed that osteogenic differentiation was downregulated significantly by 2 mM of metformin and also due to glucose restriction. This occurrence was associated by the authors with AMPK activity, as its phosphorylation was induced by metformin. Additionally it was proven that sustained phosphorylation of AMPK was well correlated with the inhibition of matrix mineralization. The authors of this paper revealed that phosphorylation of AMPKa in osteoblasts caused by metformin strongly depends on its dose, which is very important.

The metformin action in time- and dose-dependent manner was also noticed by the other researcher groups in various cell cultures. The concentrations of metformin which was used in this study were also tested in cultures of human oral squamous cell carcinoma and significantly reduced the colony formation of those cells* in vitro* [6]. Dose related effects of metformin were also investigated using stromal cells. Recently Abu-Zaiton [46] has shown that metformin at concentration of 100 *μ*g/mL inhibits fibroblast proliferation, at both the normoxia and hypoxia conditions. Gao et al. [52] showed also that metformin may negatively influence adipogenic differentiation of progenitor cells isolated from bone marrow. Interestingly, the effects of metformin on stromal cells were tested at concentrations lower than doses used in experiments showing antitumor effect of this drug [46, 52, 53]. The antitumor effect of metformin investigated* in vitro* was established for doses from 5 to 30 mM [1, 4–6, 54]. These concentrations of metformin tested on cancer cell cultures are considered as well above the concentration in which metformin can be safely used* in vivo* [30, 55]. But another point of view was stated by Wilcock and Bailey [31]. It was shown that the levels of metformin that accumulate in the tissues might be several times higher than in the blood, and thus metformin acts at a significantly higher level in the target organs.

Additionally, results of microscopic evaluation of ASCs morphology and ultrastructure have shown that high doses of metformin (5 mM and 10 mM), which were used for* in vitro* studies, possess a cytotoxic effect on the cells and may cause (i) disintegration of cells, (ii) nucleus fragmentation and production of apoptotic bodies, (iii) enlargement of mitochondria, (iv) inhibition of MVs shedding, and (v) limited development of cellular projections. To the best of our knowledge, this is the first report describing morphological and ultrastructural changes of ASCs under the influence of metformin. However the observations concerning ultrastructural alterations resulting from the metformin action are consistent with the descriptions presented by Pounaghi et al. [56] who have noticed shape shrinking and size decreasing of granulosa cells, as well as nuclei shrinking and reduction in number of cytoplasmic organelles and their unnatural appearance. Interestingly, the enlargement of mitochondria observed in ASCs treated with 5 mM of metformin is a feature which was noticed also in granulosa cells and nontransformed breast epithelial cells [56, 57].

Kiefer et al. [58] have noticed that osteopontin is strongly upregulated in adipose tissue of high-fat diet-induced and genetically obese mice. Our results show that osteopontin expression in adipose tissue of mice treated with metformin decreased in comparison with control group, not receiving metformin. The decrease of tissue OPN expression was correlated with the decrease of other investigated markers of proliferating cells, that is, Ki-67 and CD105. Both Ki-67 and CD105 are proteins essential for cells proliferation [59]. The downregulation of Ki-67 expression resulting from metformin treatment was noted previously in breast and ovarian tumors [60]. Ki-index is highly correlated with cancer progression as it is strongly expressed by proliferating cancer cells. Recently, Tebbe et al. [60] have shown that metformin limits adipocyte mediated ovarian cancer cell proliferation, migration, and expression of cancer associated genes and bioenergetic changes. The inhibition of adipocytes proliferative potential was associated with inhibition of Ki-67 expression in differentiating adipocytes. Paper of Tebbe et al. [60] supported the thesis that metformin inhibits differentiation of murine preadipocytes, stated previously by the other authors [61, 62]. Our results are in good agreement with this postulate, because in the adipose tissue of mice treated with metformin the decrease of CD105 protein expression was noted. Moreover, after metformin treatment, we have observed the increased expression of caspase-3 in adipose tissue biopsies. As it was previously shown, inhibitory effect of metformin on cancer cells may be the result of activation of caspase-dependent apoptosis [63, 64]. Therefore, obtained results of immunostaining assay show that metformin not only may inhibit self-renewal properties of adipocytes, but also may induce their death.

Additionally, in our model, the decrease of tissue OPN expression after metformin treatment correlated also with decreased levels of this protein in mice serum. Number of human studies reported elevated circulating OPN levels in obese individuals compared with lean subjects [50]. Surprisingly, results presented by Kiefer et al. [58] and You et al. [65] showed that circulating levels of OPN do not correspond with OPN expression in adipose tissue of individuals with diet-induced obesity, in both mice and humans. Results of Kiefer et al. [58] and You et al. [65] indicate that local concentrations of OPN in adipose tissue may not affect its systemic levels. Based on the results obtained in our model, it can be concluded that metformin regulates both local and systemic OPN levels.

Our experiment also showed the effect of metformin on an adipocytes size. Morphometric correlation of adipocytes size from adipose tissue of control and experimental group revealed that metformin treatment significantly decreased diameter and area of adipocytes. This observation is in a good agreement with Ciaraldi et al. [41] and Stumvoll et al. [42]. Additionally, we noted that metformin may influence adipose tissue composition, due to the fact that adipose tissue of mouse treated with metformin had more heterogeneous structure.

Quantitative evaluation of crown-like structures occurring around adipocytes revealed that macrophage infiltration into adipose tissue is similar in both investigated groups of animals. The fact that metformin may regulate body weight [11, 12] was also evident in our model. Based on the clinical characteristics of animals, metformin reduced body weight. Moreover, metformin influenced glucose and triglyceride levels causing their decrease; however, in contrary to previous reports, our results indicate elevated levels of cholesterol in group receiving metformin [66, 67].

## 5. Conclusions

In conclusion, metformin affects morphology of adipose-derived multipotent mesenchymal stem cells and adipocytes. Moreover, this compound highly influences proliferation of cells derived from adipose tissue. Targeting OPN expression and synthesis by the metformin action may significantly inhibit proliferation and differentiation of adipocytes and adipocytes precursors. Because obesity, cancer, and aging have numerous interrelationships, the recognition of metformin influence on OPN expression may bring a key that will help to tackle epidemics of obesity, diabetes, and cancer. Additionally, dose titration of metformin appears to be crucial, as effects of metformin action strongly relates to its concentration. An important part of future studies, related to metformin effects on stromal cells, should be a detailed analysis of the mechanisms of its action. As it was previously noted, the inhibitory effect of metformin on cells proliferation and differentiation may vary among cells. It seems that a strong clue that will allow for clarification of this issue might be elucidation of AMPK role in these cellular processes.

## Figures and Tables

**Table 1:** Results of clinical characterization of animals used for the experiment.

Parameters	Control group (*n* = 9)	Experimental group (*n* = 9)
Body weight (g)	19.62 ± 0.55	18.34 ± 0.53
Fasting blood glucose (mmol/L)	13.30 ± 0.43	12.70 ± 0.26
Fasting serum triglyceride (mmol/L)	0.62 ± 0.05	0.53 ± 0.03
Fasting serum cholesterol (mmol/L)	1.34 ± 0.02	1.45 ± 0.03
